# Amb a 1-Specific IgE in Heart Failure: A Translational Framework for Seasonal Risk, Endotyping, and Patient-Centered Management

**DOI:** 10.3390/jcm15155971

**Published:** 2026-07-31

**Authors:** Camelia-Felicia Bănărescu, Octavia Harich, Cristina Uța, Laura Haidar, Roxana Maria Buzan, Elena-Larisa Zimbru, Sandra Iulia Moldovan, Carmen Panaitescu, Alina Andreea Tischer, Elena Daniela Jurj, Diana-Maria Mateescu, Filip-Alin Banarescu, Virgil Păunescu

**Affiliations:** 1 Center of Immuno-Physiology and Biotechnologies, Department of Functional Sciences, “Victor Babeș” University of Medicine and Pharmacy, 2 Eftimie Murgu Square, 300041 Timisoara, Romania; felicia.banarescu@umft.ro (C.-F.B.); cristina.uta@umft.ro (C.U.); buzan.roxana@umft.ro (R.M.B.); sandra.moldovan@umft.ro (S.I.M.); cbunu@umft.ro (C.P.); vpaunescu@umft.ro (V.P.); 2 Department of General Medicine, Doctoral School, “Victor Babeș” University of Medicine and Pharmacy, Eftimie Murgu Square 2, 300041 Timisoara, Romania; 3 Physiology Discipline, Department of Functional Sciences, Faculty of Medicine, “Victor Babeș” University of Medicine and Pharmacy, 2 Eftimie Murgu Square, 300041 Timisoara, Romania; 4 Emergency Clinical Hospital “Pius Brinzeu”, 156 Liviu Rebreanu Bd., 300723 Timisoara, Romania; 5 Research Center for Gene and Cellular Therapies in the Treatment of Cancer—OncoGen, Timiș County Emergency Clinical Hospital “Pius Brinzeu”, 156 Liviu Rebreanu Bd., 300723 Timisoara, Romania; 6 Cardiovascular Disease Institute Timisoara, Gheorghe Adam St., No. 13A, 300310 Timisoara, Romania; 7 Ear-Nose-Throat Department, “Victor Babeș” University of Medicine and Pharmacy, 300041 Timisoara, Romania; tischer.alina@umft.ro; 8 Center for Drug Data Analysis, Cheminformatics, and the Internet of Medical Things, “Victor Babeș” University of Medicine and Pharmacy, Eftimie Murgu Square, No. 2, 300041 Timisoara, Romania; daniela.adam@umft.ro; 9 Department of Infectious Diseases, Timisoara Clinical Railway Hospital, 1 Gării Street, 300166 Timisoara, Romania; diana.mateescu@umft.ro; 10 Department of Infectious Diseases, “Victor Babeș” Clinical Hospital for Infectious Diseases and Pneumophthisiology, 13 Gheorghe Adam Street, 300310 Timisoara, Romania; filip.banarescu@gmail.com

**Keywords:** Amb a 1, ragweed, allergen-specific IgE, heart failure, mast cells, cardiac remodeling, HFpEF, component-resolved diagnosis, seasonal symptoms, cardio-allergology

## Abstract

**Background/Objectives**: Amb a 1 is the major allergenic component of *Ambrosia artemisiifolia* pollen and a clinically relevant marker of genuine ragweed sensitization. Heart failure is increasingly recognized as a systemic syndrome shaped by immune activation, endothelial dysfunction, fibrosis, neurohormonal imbalance, pulmonary comorbidity, and environmental exposures. This narrative review aims to synthesize the translational evidence linking Amb a 1-specific IgE, IgE-mediated inflammation, allergic airway disease, and cardiovascular remodeling in heart failure. **Methods**: A targeted narrative review was performed, integrating evidence on component-resolved ragweed diagnosis, IgE-FcεRI signaling, mast cell and eosinophil biology, pollen exposure, cardiovascular inflammation, and heart failure pathophysiology. **Results**: No dedicated clinical studies have validated Amb a 1-specific IgE as a diagnostic, prognostic, or therapeutic biomarker in heart failure. However, adjacent evidence supports biologically plausible links between allergen-specific IgE responses and cardiovascular dysfunction, including mast cell activation, cytokine release, endothelial perturbation, oxidative stress, microvascular dysfunction, pulmonary-cardiac interaction, and myocardial fibrosis. Amb a 1-specific IgE may therefore identify a seasonally vulnerable heart failure phenotype, particularly in patients with allergic rhinitis, asthma, eosinophilic inflammation, or recurrent symptom worsening during ragweed season. A systemic/indirect pathway operating through allergic airway disease is distinguished from a postulated direct cardiac pathway; the latter remains strictly speculative, as no direct evidence demonstrates that inhaled Amb a 1 reaches or activates cardiac mast cells in vivo. **Conclusions**: Amb a 1-specific IgE should not currently be used to infer cardiac causality or modify heart failure therapy. Prospective, phenotype-rich, exposure-informed studies are needed to determine whether ragweed sensitization has clinically meaningful implications for heart failure endotyping, seasonal risk assessment, and cardio-allergology care. These findings may inform patient-centered heart failure management by improving the interpretation of seasonal dyspnea, allergic comorbidity, and symptom fluctuations in ragweed-endemic regions.

## 1. Introduction

Heart failure (HF) is a clinical syndrome rather than a single disease entity. Contemporary European and North American documents define and classify HF using symptoms and signs, natriuretic peptides, structural or functional cardiac abnormalities, and left ventricular ejection fraction (LVEF) categories, including HF with reduced ejection fraction (HFrEF), mildly reduced ejection fraction (HFmrEF), preserved ejection fraction (HFpEF), and improved ejection fraction (HFimpEF) [1,2,3,4]. Despite major progress in guideline-directed medical therapy, HF remains highly heterogeneous. Patients with similar LVEF may differ substantially in inflammatory burden, fibrosis, pulmonary comorbidity, obesity, renal dysfunction, autonomic tone, and environmental vulnerability. This heterogeneity has encouraged endotyping approaches that seek biologically coherent subgroups with distinct symptom trajectories, biomarker patterns, decompensation triggers, and therapeutic opportunities.

Inflammation and extracellular matrix remodeling are central to this endotyping perspective. HFpEF has been conceptualized as a comorbidity-driven syndrome in which systemic inflammation promotes coronary microvascular dysfunction, cardiomyocyte stiffness, and myocardial fibrosis; HFrEF also involves inflammatory activation during myocardial injury, repair, and adverse remodeling [5,6,7,8,9,10]. The immunoglobulin E (IgE) axis intersects with this biology. Experimental and clinical data suggest that total IgE and IgE-FcεRI signaling may participate in pathological cardiac remodeling, cardiac fibrosis, atherogenesis, and cardiovascular mortality, while newer work indicates that HF itself may influence IgE biology through spleen–heart immune communication and CD23-mediated pathways [11,12,13,14,15,16,17]. Cardiac mast cells provide an additional link between allergic effector biology and cardiovascular remodeling because they can release vasoactive, proteolytic, profibrotic, and inflammatory mediators in the myocardium, vasculature, and right-ventricular–pulmonary interface [18,19,20,21].

Molecular allergology offers a more precise framework than crude extract testing. Component-resolved diagnosis (CRD) can distinguish genuine sensitization from cross-reactivity and refine clinical interpretation in polysensitized patients [22,23,24]. In ragweed allergy, Amb a 1 is the dominant major allergen and accounts for a large proportion of ragweed-specific IgE reactivity in sensitized individuals. Ragweed pollen allergy is an important and expanding European public health problem driven by plant invasion, climate change, pollution, and regional exposure intensity. Clinical and molecular studies across Europe confirm clinically relevant ragweed rhinitis, conjunctivitis, asthma, and Amb a 1 sensitization [25,26,27,28,29,30,31,32,33]. The geographical distribution of this burden is highly uneven and merits specification. Ragweed invasion in Europe is concentrated in the Pannonian Basin, encompassing Hungary, Serbia, Croatia, Slovakia, western Romania, and parts of Ukraine, with secondary foci in the Rhône Valley of France and the Po Valley of northern Italy [25,26,27]. Sensitization rates track this distribution closely, ranging from below 2.5% in Finland to above 50% in established centers of infestation such as Szeged and Budapest in Hungary, with rates exceeding 50% also reported from parts of Serbia, Croatia, Bulgaria, and northern Italy and intermediate values in France and Austria [25,26]. Estimates for Europe as a whole indicate that approximately 13.5 million people are affected by ragweed allergy, a figure projected to rise with continued climate-driven range expansion [26,27]. Romania lies within the Pannonian focus, and the western part of the country in particular constitutes one of the most heavily exposed regions. In a pollen-exposed cohort from northern Italy, 66 Amb a 1-allergic participants showed significant associations between airborne ragweed pollen concentrations and daily ocular, nasal, and bronchial symptom severity [34]. Component-resolved studies in European cohorts have demonstrated IgE reactivity to Amb a 1 together with cross-reactive profilins, polcalcins, and homologous weed-pollen allergens [28,29,35]. This regional concentration is central to the argument of the present review: the exposure-informed studies proposed below are feasible only in populations where both ragweed sensitization and heart failure are sufficiently prevalent for their overlap to be studied, and western Romania and the wider Pannonian Basin represent precisely such settings. Environmental and epidemiological studies also suggest that pollen exposure and allergic airway disease may intersect with cardiovascular outcomes, although the available findings are heterogeneous and often confounded by heat, air pollution, respiratory infections, and behavioral factors [36,37,38,39,40,41,42,43].

The central knowledge gap is therefore specific and clinically testable: Amb a 1-specific IgE is well established in ragweed molecular allergology, and the IgE–mast cell axis is increasingly relevant to cardiovascular remodeling, but Amb a 1-specific IgE has not been directly validated as a biomarker or endotyping tool in HF. This review addresses that gap by synthesizing the evidence needed to define a conservative translational framework for future clinical studies. The aim is not to imply causality but to identify when Amb a 1-specific IgE may be clinically informative, what it should not be used to conclude, and how future HF studies should measure allergy-related and season-related variables without overinterpretation. In line with contemporary heart failure management priorities, this review also emphasizes the patient-centered implications of seasonal allergic burden. In clinical practice, dyspnea, fatigue, reduced exercise tolerance, sleep disruption, and recurrent healthcare contact may reflect overlapping cardiac, pulmonary, allergic, and environmental drivers. A cardio-allergology perspective may therefore support more precise symptom interpretation, avoid unnecessary escalation of heart failure therapy, and improve individualized seasonal prevention strategies in ragweed-endemic regions.

## 2. Materials and Methods

This article was designed as a narrative and translational review rather than a systematic review or meta-analysis. The objective was to integrate evidence across several partially overlapping domains: HF phenotyping and remodeling, IgE- and FcεRI-mediated cardiovascular mechanisms, mast cell and basophil biology, molecular allergology, ragweed/Amb a 1 sensitization, allergic airway disease, and pollen-related cardiovascular epidemiology.

A structured narrative search strategy was used to identify relevant literature in PubMed/MEDLINE, Scopus, Web of Science, and Google Scholar. The principal literature search covered the period from database inception to 30 November 2025 and was conducted between 1 September 2025 and 30 November 2025. A verification search was performed on 15 January 2026, and additional targeted searches were conducted during peer-review revision to capture newly published records directly relevant to the reviewers’ requests, including 2025–2026 studies on inflammatory HF phenotypes, right-ventricular dysfunction, and regional ragweed exposure. No language restriction was applied at the search stage. Full texts available in English or Romanian were assessed directly; non-English publications with sufficiently detailed English-language abstracts and verified bibliographic information were considered when directly relevant to the review question. Searches combined terms related to “heart failure”, “HFpEF”, “HFrEF”, “cardiac remodeling”, “cardiac fibrosis”, “IgE”, “FcεRI”, “CD23”, “mast cells”, “basophils”, “eosinophils”, “*Ambrosia artemisiifolia*”, “ragweed”, “Amb a 1”, “component-resolved diagnosis”, “molecular allergology”, “allergic rhinitis”, “asthma”, “pollen exposure”, “cardiovascular morbidity”, and “blood pressure”. Additional references were identified by screening the reference lists of key guidelines, mechanistic reviews, and original articles. The search prioritized clinical guidelines, mechanistic studies with cardiovascular relevance, molecular allergology papers, epidemiological studies of pollen and cardiovascular outcomes, and Romanian or Central/Eastern European data on ragweed sensitization.

The review included human studies, experimental studies, clinical guidelines, mechanistic reviews, and translational articles considered relevant to the proposed interface between Amb a 1-specific IgE and HF biology. Publications were excluded when they were not relevant to HF, IgE/allergic inflammation, ragweed molecular sensitization, or environmental cardiovascular risk. Because this was not a systematic review, no formal risk-of-bias tool, meta-analysis, or evidence grading was applied. Instead, the synthesis explicitly distinguishes direct evidence, mechanistic plausibility, clinical inference, and unresolved research questions. At the time of manuscript preparation, no dedicated clinical cohort study was identified that directly evaluated Amb a 1-specific IgE in patients with established HF. Consequently, the manuscript is framed as a hypothesis-generating review and avoids diagnostic, prognostic, or therapeutic claims that are not supported by direct HF data. Because the review was intended for a heart failure management context, particular attention was given to studies and concepts relevant to symptom interpretation, patient-reported dyspnea, quality of life, comorbidity management, seasonal prevention, and multidisciplinary care.

## 3. Evidence Map: What Is Known and What Remains Inferential

The available evidence is best interpreted as a set of adjacent literatures rather than as a single validated clinical pathway. Table 1 separates established background evidence from mechanistic and inferential links. This distinction is essential because findings concerning total IgE, mast cells, or allergic airway disease cannot be automatically extrapolated to Amb a 1-specific IgE in HF patients.

Because no study has yet examined Amb a 1-specific IgE in heart failure, it is useful to identify explicitly which existing studies come closest to the question and precisely where each falls short. Two are worth singling out.

The first is the Atlanta time-series analysis by Lappe and colleagues, which examined daily concentrations of thirteen pollen taxa in relation to emergency department visits for eight cardiovascular outcomes over a 26-year period and included more than 200,000 visits for congestive heart failure among more than 1.5 million cardiovascular presentations [38]. This is the largest identified cardiovascular dataset in which congestive heart failure presentations were evaluated against ambient pollen concentrations. However, the investigators found predominantly null associations and concluded that the evidence for an effect of pollen on cardiovascular morbidity was limited. Its limitations with respect to the present question are decisive: exposure was defined aerobiologically by pollen taxon rather than immunologically, individual sensitization status was unknown, and ragweed was analyzed as one taxon among many rather than resolved to the Amb a 1 molecular level. The study therefore cannot distinguish an allergen-specific mechanism in sensitized individuals from a non-specific seasonal effect in the general population.

The second is the analysis of Al-Mukhtar and colleagues, which related temporal changes in pollen concentration to short-term clinical outcomes in acute coronary syndromes and reported associations between pre-admission pollen exposure and in-hospital outcomes, with inflammatory activation proposed as the mediating mechanism [37]. Its relevance here is mechanistic rather than direct: it supports the plausibility of a pollen cardiovascular signal acting through inflammation, but the outcome was acute coronary syndrome rather than heart failure, the exposure was again aerobiological rather than sensitization-defined, and no component-resolved testing was performed.

Taken together, these two studies define both the current ceiling of the evidence and the specific gap addressed by this review. Al-Mukhtar et al. reported associations between short-term changes in pollen concentrations and selected acute coronary syndrome presentations and outcomes, whereas Lappe et al. found predominantly null associations and only limited evidence linking pollen exposure with cardiovascular morbidity. Neither study assessed individual sensitization, ragweed-specific IgE, or Amb a 1. Accordingly, neither can determine whether a cardiovascular signal is allergen-specific or confined to sensitized individuals. This is why the interaction between pollen exposure and Amb a 1-specific IgE status, rather than a simple seasonal association, is proposed in Section 8.3 as the primary estimand for future work.

## 4. Amb a 1 and Component-Resolved IgE Testing: Why the Specific Molecule Matters

Crude ragweed extract IgE is clinically useful but analytically heterogeneous. Extracts contain multiple allergenic and non-allergenic proteins, and extract positivity may reflect genuine sensitization, co-sensitization, profilin-mediated cross-reactivity, calcium-binding proteins, cross-reactive carbohydrate determinants (CCDs), or variable extract composition. CRD addresses this limitation by quantifying IgE directed against defined allergenic molecules. For ragweed, Amb a 1 is the principal molecular marker of genuine sensitization, while components such as Amb a 8, Amb a 9, Amb a 10, and Amb a 11 may refine interpretation in complex or polysensitized patients [22,23,24,25,26,27,28,29,30,31,32,33].

This distinction is particularly important in HF research. A study based only on crude ragweed extract IgE may misclassify cross-reactive sensitization as clinically relevant ragweed allergy. A study based only on total IgE cannot determine whether the IgE signal reflects ragweed, other aeroallergens, food sensitization, smoking-related immune activation, parasitic exposure, atopic dermatitis, or other immune states. Amb a 1-specific IgE is therefore the most defensible molecular anchor for a cardio-allergology study in ragweed-endemic settings. However, it should not be interpreted in isolation. A robust HF study should also record total IgE, ragweed extract IgE, relevant cross-reactive components, allergic rhinitis and asthma phenotype, medication use, eosinophil count, and timing relative to pollen season.

Discordant results between crude extract and component testing are common in practice and require a structured interpretive approach, which is directly relevant to multidisciplinary cardio-allergology teams. The most frequent discordant pattern is ragweed extract positivity with Amb a 1 negativity. This combination argues against genuine ragweed sensitization and should prompt testing for cross-reactive molecules rather than a diagnosis of ragweed allergy. In such patients, IgE reactivity is frequently directed against the profilin Amb a 8, the polcalcins Amb a 9 and Amb a 10, the defensin-like molecule Amb a 4, the non-specific lipid transfer protein Amb a 6, or cross-reactive carbohydrate determinants, all of which are broadly cross-reactive across botanically unrelated pollens and plant foods and generally associated with weaker or absent clinical relevance [22,23,24,28,29]. Recognizing this pattern matters clinically because such patients are usually poor candidates for ragweed allergen immunotherapy, which targets Amb a 1-dominant responses, and attributing seasonal dyspnea to ragweed in an Amb a 1-negative patient risks overlooking a genuine cardiac or non-allergic respiratory cause of symptom worsening.

A practical sequential approach can be recommended. When crude extract IgE is positive, Amb a 1 should be quantified first. If Amb a 1 is positive, genuine sensitization is confirmed, and clinical relevance should then be established by correlating symptoms with local pollen calendars. If Amb a 1 is negative, profilin, polcalcin, and cross-reactive carbohydrate determinant markers should be assessed; positivity for these markers in a polysensitized patient generally indicates cross-reactivity rather than primary ragweed disease. In patients co-sensitized to pan-allergens, sensitization to mugwort (Art v 1), birch (Bet v 1), and grass (Phl p 1 and Phl p 5), major allergens should also be characterized, since overlapping late-summer and autumn flowering periods in Central and Eastern Europe make symptom-based attribution unreliable. Where component testing remains inconclusive but clinical suspicion is high, nasal or conjunctival allergen provocation or basophil activation testing may resolve the discordance in specialist centers, although provocation testing requires particular caution in patients with advanced HF, uncontrolled asthma, or hemodynamic instability. Conversely, Amb a 1 positivity in a patient without seasonal symptoms denotes sensitization only and should not be reported as ragweed allergy. Applying this algorithm allows cardio-allergology teams to reserve allergy-directed seasonal interventions for patients in whom a genuine, molecularly confirmed ragweed response plausibly contributes to symptom burden.

## 5. IgE–FcεRI Signaling and Cardiac Remodeling

The strongest mechanistic argument for studying Amb a 1-specific IgE in HF comes from the broader IgE–cardiovascular literature. In experimental cardiac disease models, IgE–FcεRI signaling has been associated with pathological remodeling and dysfunction, while elevated IgE has been linked to cardiac fibrosis through suppression of miR-486a-5p and downstream profibrotic signaling [11,12]. The CD23-mediated spleen–heart axis adds a second layer of relevance: HF may not simply be affected by IgE biology; HF-related immune remodeling may also influence IgE production and B-cell regulation [13]. These findings do not prove that Amb a 1-specific IgE worsens HF, but they make the hypothesis biologically testable.

The distinction between total IgE and allergen-specific IgE is central. Total IgE may behave as a systemic immune marker, whereas Amb a 1-specific IgE identifies a defined antigenic memory against ragweed. In an Amb a 1-sensitized HF patient, two mechanistically distinct pathways must be separated explicitly because they differ fundamentally in the strength of the evidence supporting them and their testability.

Pathway A (systemic/indirect pathway). Inhaled Amb a 1 elicits IgE-mediated inflammation within the upper and lower airways, producing allergic rhinitis and asthma exacerbation. The resulting nasal obstruction, nocturnal cough, sleep fragmentation, intermittent hypoxemia, sympathetic surge, dynamic hyperinflation, and reduced physical activity impose secondary hemodynamic and mechanical stress on the heart, particularly on the right ventricle. Each individual step of this pathway is documented in the respiratory and cardiovascular literature, and the pathway is therefore empirically tractable in HF cohorts using established respiratory, polysomnographic, and hemodynamic measurements.

Pathway B (direct cardiac pathway). Amb a 1-mediated activation of cardiac tissue-resident mast cells or intramyocardial basophils causes localized mediator release, with consequent coronary microvascular dysfunction, fibroblast activation, and extracellular matrix remodeling. It must be stated unambiguously that Pathway B remains strictly speculative and lacks direct experimental validation. Although total IgE and cardiac tissue-resident mast cells have been linked to myocardial fibrosis in experimental models, there is at present no direct empirical evidence that an inhaled aeroallergen such as Amb a 1 reaches the myocardial interstitium in immunologically meaningful quantities, binds FcεRI on cardiac mast cells in vivo, or triggers their degranulation in the intact human heart. The transfer of intact allergenic protein from the airway lumen to the cardiac interstitium has never been characterized, and no animal model has demonstrated allergen-specific cardiac mast cell activation following physiological aeroallergen challenge. Pathway B is therefore presented in this review solely as a hypothesis requiring dedicated experimental testing, and no clinical inference should be drawn from it.

Throughout this review, the mechanistic evidence supporting Pathway A is treated as documented but indirect with respect to HF outcomes, whereas Pathway B is treated as unvalidated. Where the text refers to IgE-mediated effects on the cardiac microenvironment, this denotes total IgE and mast cell biology derived from experimental models and does not constitute evidence of Amb a 1-specific cardiac activation. This framework is plausible but unproven. Causality would require longitudinal data showing that Amb a 1-specific IgE-positive HF patients have distinct seasonal trajectories in symptoms, natriuretic peptides, congestion, echocardiography, hospitalization, or functional status after adjustment for asthma, chronic obstructive pulmonary disease (COPD), pollution, viral infections, medication adherence, renal function, obesity, and baseline HF severity.

**Figure 1:**
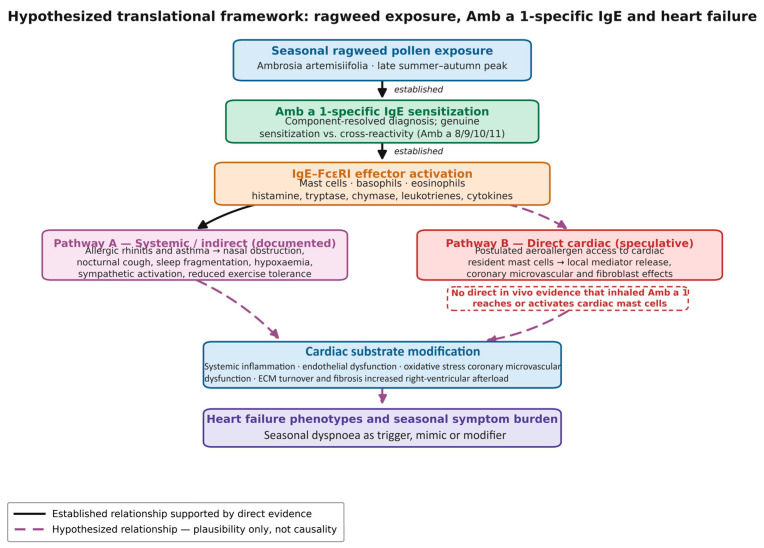
Translational framework linking ragweed exposure, Amb a 1-specific IgE, IgE-responsive effector pathways, allergic respiratory burden, and heart failure phenotypes. Line style encodes evidence level and must be read together with the legend: solid arrows denote established relationships supported by direct evidence (seasonal pollen exposure leading to Amb a 1-specific sensitization and sensitization leading to IgE-FcεRI effector activation); dashed arrows denote hypothesized relationships for which current evidence supports plausibility only and not clinical causality. All steps connecting allergen sensitization to myocardial remodeling and clinical heart failure endpoints are shown as dashed arrows. Pathway A represents the systemic/indirect route operating through allergic airway disease; Pathway B represents the postulated direct cardiac route, which is explicitly flagged within the figure as lacking direct in vivo evidence and is strictly speculative. The model is hypothesis-generating and does not establish causality. Abbreviations: ECM, extracellular matrix; FcεRI, high-affinity IgE receptor; HFpEF, heart failure with preserved ejection fraction; HFrEF, heart failure with reduced ejection fraction.

The proposed translational pathway is presented in Figure 1, which summarizes the hypothesized sequence linking ragweed exposure, Amb a 1-specific IgE, IgE-responsive effector pathways, allergic respiratory burden, and heart failure phenotypes.

## 6. Mast Cells, Basophils, Eosinophils, and the Cardiac Microenvironment

Mast cells are long-lived tissue-resident immune cells located in the myocardium, perivascular spaces, atherosclerotic plaques, valves, and perineural regions. Their mediator repertoire includes histamine, tryptase, chymase, leukotrienes, prostaglandins, cytokines, growth factors, matrix metalloproteinases, and profibrotic signals [18,19,20,21]. In allergic disease, allergen-specific IgE binds to FcεRI on mast cells and basophils permits rapid activation after antigen exposure. In HF, the same effector compartment could theoretically influence vascular permeability, coronary microvascular tone, endothelial activation, fibroblast behavior, extracellular matrix turnover, and pulmonary vascular load.

Basophils and eosinophils add further complexity. Basophils may reflect systemic type 2 immune activation and can be evaluated using basophil activation tests in research settings. Eosinophils are common in allergic airway disease and can contribute to tissue inflammation, but common allergic sensitization must not be conflated with eosinophilic myocarditis or hypereosinophilic syndromes. For clinical HF research, the most defensible approach is to embed Amb a 1-specific IgE in a multidimensional panel that includes total IgE, allergen-specific IgE, eosinophils, rhinitis/asthma phenotype, pulmonary function, natriuretic peptides, troponin, renal function, echocardiography, and seasonal exposure.

**Figure 2:**
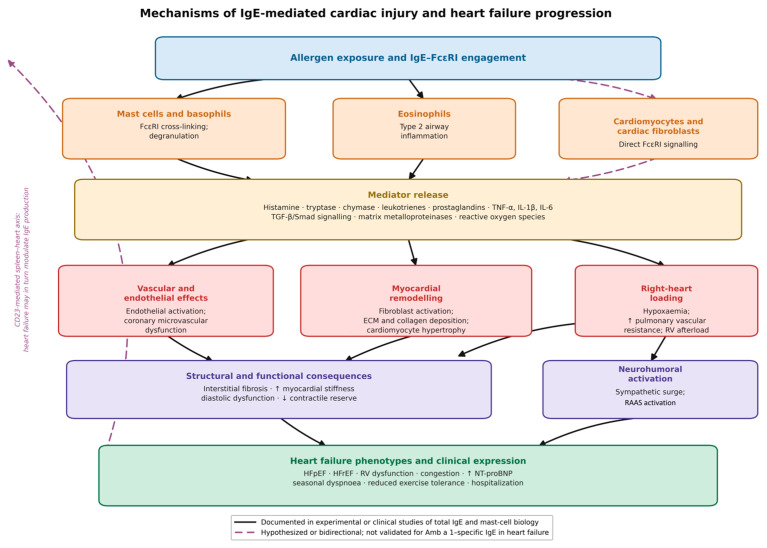
Mechanisms of IgE-mediated cardiac injury and heart failure progression. Allergen exposure and IgE–FcεRI engagement activate mast cells, basophils, and eosinophils and may act directly on cardiomyocytes and cardiac fibroblasts. The resulting mediators, including histamine, tryptase, chymase, leukotrienes, pro-inflammatory cytokines, TGF-β/Smad signaling, matrix metalloproteinases, and reactive oxygen species, promote endothelial activation and coronary microvascular dysfunction, fibroblast activation with extracellular matrix deposition and cardiomyocyte hypertrophy, and increased right-ventricular loading through hypoxemia and raised pulmonary vascular resistance. These processes converge on interstitial fibrosis, increased myocardial stiffness, diastolic dysfunction, and reduced contractile reserve, which, together with neurohumoral activation, determine the clinical heart failure phenotype. The dashed arrow on the left denotes the CD23-mediated spleen–heart axis, by which established heart failure may, in turn, modulate IgE production. As in Figure 1, line style encodes evidence level: solid arrows denote steps documented in experimental or clinical studies of total IgE and mast cell biology, whereas dashed arrows denote hypothesized or bidirectional relationships that have not been validated for Amb a 1-specific IgE in heart failure. The figure depicts general IgE-related mechanisms and must not be read as evidence of allergen-specific cardiac activation. Abbreviations: ECM, extracellular matrix; FcεRI, high-affinity IgE receptor; HFpEF, heart failure with preserved ejection fraction; HFrEF, heart failure with reduced ejection fraction; IL, interleukin; RAAS, renin–angiotensin–aldosterone system; RV, right ventricle; TGF-β, transforming growth factor beta; TNF-α, tumor necrosis factor alpha. The upward arrow (↑) indicates an increase, whereas the downward arrow (↓) indicates a decrease.

The mechanisms outlined above can be represented as a staged sequence linking allergen-driven IgE–FcεRI engagement to the structural and functional abnormalities that characterize heart failure. Figure 2 summarizes this pathogenetic cascade, again distinguishing steps supported by experimental or clinical evidence from those that remain hypothesized for Amb a 1-specific IgE.

## 7. Clinical Interpretation of Amb a 1-Specific IgE in HF Patients

Table 2 summarizes defensible interpretations of common testing scenarios. The key principle is that Amb a 1-specific IgE indicates molecular sensitization; it does not, by itself, diagnose allergic disease activity, HF decompensation, cardiac remodeling, or treatment response.

## 8. Translational Relevance and Research Priorities

### 8.1. Why This Topic Is Clinically Relevant Despite Limited Direct Evidence

The topic is clinically relevant when framed as an endotyping hypothesis rather than an established biomarker claim. HF care already recognizes that dyspnea, fatigue, exercise intolerance, congestion, and hospitalization are shaped by extracardiac systems, particularly the lungs, kidneys, vasculature, autonomic nervous system, and immune system. Ragweed allergy is common in several European regions, including Romania; Amb a 1-specific IgE is measurable using standardized molecular diagnostics; and IgE-responsive immune pathways have plausible links to remodeling and symptoms. The unmet need is not another broad inflammatory marker, but a more precise way to test whether an aeroallergen-specific immune signal modifies HF phenotype, seasonal symptom instability, or cardio-pulmonary comorbidity patterns.

### 8.2. Amb a 1-Specific IgE as an Atopic HF Endotype Marker

The most defensible clinical role of Amb a 1-specific IgE is endotyping. In an HF clinic, patients with similar LVEF and NT-proBNP can have very different causes of dyspnea. A patient with HFpEF, obesity, hypertension, rhinitis, and ragweed asthma may experience seasonal dyspnea from airway obstruction, nocturnal symptoms, reduced sleep quality, sympathetic activation, or reduced physical activity. Another patient with HFrEF and no allergic airway disease may have dyspnea driven predominantly by congestion or low cardiac output. Amb a 1-specific IgE could help identify the first phenotype, particularly when interpreted together with symptoms and pollen season. This is clinically relevant because uncontrolled allergic airway disease may lead to avoidable medication changes, unnecessary escalation of diuretics, or misclassification of respiratory symptoms as HF decompensation.

#### Relationship to Inflammatory Phenotypes of Heart Failure

This endotyping proposal should be positioned within the wider movement toward phenotype-based classification of heart failure, which provides both the conceptual justification for the approach and a concrete methodological template. Contemporary heart failure research increasingly holds that ejection fraction alone is an insufficient organizing principle and that biologically defined subgroups cutting across ejection fraction categories may better reflect underlying pathophysiology.

The network analysis of Tromp and colleagues is instructive in this regard. Using 92 biomarkers spanning multiple pathophysiological domains in 1544 patients with heart failure, with independent validation in a further 804, the authors derived biological pathways uniquely associated with HFrEF, HFmrEF, and HFpEF [44]. Inflammatory and immune-related pathways emerged as differentially represented across the ejection fraction spectrum, supporting the view that inflammation is not a uniform feature of heart failure but is concentrated in identifiable subgroups. The methodological implication for the present hypothesis is direct: if inflammatory activation is heterogeneously distributed across heart failure phenotypes, then any allergen-driven immune contribution would likewise be expected to concentrate within particular phenotypes rather than act uniformly, and analyses that pool all heart failure patients would dilute such an effect toward the null.

Work on the phenotypic subdivision of HFpEF reinforces this point. Plotnikova and colleagues reviewed current concepts of HFpEF phenotyping and emphasized the substantial pathophysiological heterogeneity of the syndrome, including proposed inflammatory, fibrotic, and dysmetabolic phenotypes [45]. Since HFpEF is proposed above as the most informative setting for early studies, this internal heterogeneity is directly relevant: an atopic contribution, if present, would be expected within an inflammatory or inflammatory–metabolic subgroup rather than within predominantly hypertensive, ischemic, or infiltrative HFpEF phenotypes.

An Amb a 1-defined atopic subgroup could therefore be conceived of as a candidate seasonal modifier of an existing inflammatory phenotype rather than a new phenotype in its own right. Two implications follow for study design. First, inflammatory phenotyping should be performed at baseline, using established markers such as C-reactive protein, interleukin-6, tumor necrosis factor alpha, galectin-3, and soluble ST2, so that Amb a 1-sensitized patients can be positioned within rather than alongside the inflammatory landscape of the cohort. Second, the analysis should test whether sensitization is enriched within, or interacts with, a pre-existing inflammatory phenotype, rather than treating atopy as an independent axis. It must be emphasized, however, that neither of these studies examined allergen sensitization, and the connection drawn here is an inference about study architecture rather than evidence that ragweed sensitization contributes to any established inflammatory phenotype.

### 8.3. Seasonal Decompensation: Trigger, Mimic, or Modifier?

Seasonal worsening in HF can result from many overlapping exposures: heat, humidity, air pollution, respiratory viruses, dietary changes, medication adherence, travel, renal function shifts, and pollen. Ragweed season adds a plausible allergic burden in sensitized patients. Importantly, ragweed exposure may act in three different ways. First, it may be a trigger, meaning that it contributes directly to worsening dyspnea or healthcare use. Second, it may be a mimic, meaning that allergic rhinitis or asthma symptoms resemble HF worsening without actual congestion. Third, it may be a modifier, meaning that it does not initiate decompensation but lowers the threshold at which cardiac stress becomes symptomatic. Future studies should separate these possibilities by combining patient-reported symptoms, physical congestion assessment, NT-proBNP dynamics, lung function, and exposure data. A single cross-sectional IgE measurement cannot distinguish these mechanisms.

A specific methodological obstacle deserves emphasis because it determines whether any future trial in this area can produce interpretable results. The ragweed season in Central and Eastern Europe, spanning late summer and early autumn, coincides with a dense cluster of independent cardiovascular stressors. Extreme heatwaves, abrupt temperature drops at the summer–autumn transition, elevated concentrations of particulate matter (PM_2.5_ and PM_10_), nitrogen dioxide, and ground-level ozone, and the onset of seasonal respiratory viral circulation are each well-established, independent drivers of HF hospitalization, dyspnea, and cardiovascular mortality [36,38]. Heat stress precipitates decompensation through dehydration, hemoconcentration, diuretic-related electrolyte disturbance, and reflex tachycardia; particulate matter promotes systemic inflammation, endothelial dysfunction, and autonomic imbalance; and respiratory infections are among the commonest precipitants of acute HF. Because all of these exposures rise and fall on partly overlapping seasonal timescales, ragweed pollen concentration is strongly collinear with atmospheric and meteorological confounders. Any study that regresses HF outcomes on pollen counts without explicit adjustment will therefore recover an uninterpretable composite of seasonal exposures rather than an Amb a 1 signal.

Several epidemiological and statistical strategies can isolate the allergen-specific component. First, time-stratified case-crossover designs are the preferred primary analysis because each patient serves as their own control and all time-invariant confounders, including age, sex, HF phenotype, comorbidity, socioeconomic status, and baseline medication, are eliminated by design; referent days should be selected within the same calendar month and matched on day of the week to control for long-term trend, season, and weekly healthcare utilization cycles. Second, distributed lag non-linear models should be fitted over a lag window of at least 0 to 14 days, since the effects of pollen, temperature, and particulate matter on cardiovascular events follow different and non-linear lag structures; simultaneous inclusion of pollen, apparent temperature, PM_2.5_, and ozone terms allows their lag surfaces to be separated. Third, models must adjust for daily mean and apparent temperature using natural cubic splines, relative humidity, barometric pressure, PM_2.5_, PM_10_, NO_2_, and maximum 8 h ozone and should include a time-varying offset for local respiratory viral activity derived from sentinel influenza-like illness or SARS-CoV-2 surveillance data, together with indicator terms for public holidays.

Fourth, and most importantly, the design should exploit sensitization status as an internal control. The critical test is not whether HF events increase during ragweed season, which would be uninterpretable, but whether the association between pollen concentration and HF outcomes differs significantly between Amb a 1-specific IgE-positive and IgE-negative patients. This interaction term is the primary estimand because meteorological and pollution confounders act on both strata approximately equally, whereas a genuine allergen effect should be confined to, or substantially stronger in, the sensitized stratum. A formal test for effect modification by sensitization status therefore provides a confounder-resistant estimate that a simple seasonal comparison cannot. Fifth, negative control exposures should be prespecified, for example, grass or birch pollen concentrations outside their respective flowering periods, and negative control outcomes unrelated to allergic or cardiac mechanisms, such as traumatic admissions, should be analyzed to detect residual seasonal confounding. Sixth, sensitivity analyses should model pollen both as continuous exposure and using clinically established threshold categories, restrict analysis to days without heatwave alerts or air quality exceedances, and apply quantitative bias analysis or E-value calculation to quantify the strength of unmeasured confounding that would be required to nullify an observed association. Finally, exposure misclassification should be reduced by using patient-level or geocoded pollen estimates rather than a single regional monitoring station, ideally supplemented by personal sampling or automated real-time pollen monitoring, and adjusting for time spent outdoors where such data are available. Studies that omit these safeguards should be interpreted as hypothesis-generating only.

#### Reconciling Seasonal Exposure with a Chronic Disease That Peaks in Winter

A legitimate objection to the entire framework must be addressed directly. Ragweed exposure is sharply seasonal, confined in Central and Eastern Europe to roughly six weeks between mid-August and late September, whereas chronic heart failure is a lifelong condition whose decompensations and hospitalizations characteristically peak in winter. If ragweed exposure and heart failure exacerbation peak at different times of year, how can the former be relevant to the latter?

Three considerations reconcile this apparent mismatch, and each carries a distinct testable prediction.

First, the winter predominance of heart failure hospitalization is precisely why a late-summer signal would be informative rather than redundant. Winter decompensation is driven by well-characterized factors: respiratory viral infection, cold-induced vasoconstriction and increased afterload, influenza and pneumococcal illness, reduced physical activity, and dietary and adherence changes around the holiday period. These are already recognized and already targeted by vaccination and anticipatory care. A late-summer burden of symptoms occurring outside this window would fall where clinical vigilance is lowest and where seasonal explanations are least readily invoked and is therefore more likely to be misattributed. The clinical value of identifying an August-to-September component lies in its very separation from the winter peak, not in competition with it.

Second, and most importantly, the proposed relationship is predominantly one of symptom burden and symptom attribution rather than of hospitalization. As set out in Section 8.3, ragweed exposure may act as a trigger, mimic, or modifier, and the mimic and modifier roles do not require any excess of hospital admissions. A sensitized patient with HFpEF who experiences six weeks of nocturnal cough, nasal obstruction, fragmented sleep, and reduced exercise tolerance may report worsening dyspnea, attend an unscheduled clinic visit, and receive a diuretic increase without ever being admitted and without ever having been congested. Such episodes are invisible to hospitalization-based seasonality analyses, which is one reason why a late-summer allergic contribution could persist unrecognized in routine data. The corresponding prediction is that any Amb a 1-related seasonal signal should appear first in patient-reported outcomes, quality-of-life measures, unscheduled outpatient contacts, and medication changes, and only secondarily, if at all, in admission rates. Endpoint selection in future studies must reflect this.

Third, chronicity provides the mechanism by which a transient exposure could nonetheless have persistent consequences. Heart failure progresses through cumulative injury, and the trajectory is shaped by discrete events from which recovery is often incomplete. An annual six-week period of increased inflammatory and mechanical load, recurring across years, is a plausible if unproven contributor to that cumulative burden, in the same way that recurrent respiratory infections are understood to influence long-term trajectory. Two further predictions follow: any structural effect should be cumulative and therefore detectable only over multiple seasons, and post-season measurements should be compared with pre-season baselines within the same individual rather than against cross-sectional norms.

These considerations also determine the appropriate study architecture. A design in which patients are assessed immediately before the ragweed season, again at peak exposure, and again after the season has ended, with each patient serving as their own control, isolates the ragweed window from the winter viral and cold-exposure period entirely. The winter peak thus becomes an analytical advantage rather than a confounder: the two seasons are temporally separated, and a comparison restricted to the late-summer window is not contaminated by winter drivers. Should a future study observe a late-summer signal confined to Amb a 1-sensitized patients and absent in non-sensitized patients from the same clinic and climate, the seasonal mismatch that motivates this objection would become the strongest available argument that the signal is allergen-specific rather than merely seasonal.

### 8.4. HFpEF, Asthma, Obesity, and the Pulmonary–Cardiac Overlap

HFpEF may be the most informative setting for early clinical studies. HFpEF frequently overlaps with obesity, hypertension, diabetes, atrial fibrillation, sleep apnea, COPD, and asthma. Many of these comorbidities promote systemic inflammation, endothelial dysfunction, and myocardial stiffening. Allergic rhinitis and asthma may add an intermittent inflammatory and mechanical load during pollen season. In this phenotype, Amb a 1-specific IgE is unlikely to be a primary cause of HFpEF, but it may identify a modifiable contributor to symptoms and quality of life. From a clinical perspective, this could improve diagnostic precision in patients whose dyspnea is multifactorial.

### 8.5. Right-Heart and Pulmonary Vascular Vulnerability

Right-ventricular vulnerability deserves specific attention. Allergic asthma exacerbations can increase pulmonary vascular resistance through hypoxemia, airway inflammation, and dynamic hyperinflation. In patients with pulmonary hypertension, COPD/asthma overlap, or right-ventricular dysfunction, allergen-driven respiratory episodes may have greater hemodynamic consequences than in patients with isolated left-sided disease. Mast cell mediators may also participate in pulmonary vascular remodeling and right-ventricular adaptation. Recent clinical evidence from idiopathic pulmonary arterial hypertension showed that higher serum total IgE levels were associated with NT-proBNP, pulmonary and right-ventricular pressures, maladaptive right-ventricular remodeling, and worse right-ventricular function [46]. However, the interpretive distance between that observation and the subject of the present review must be stated in the strongest terms, and the finding should not be presented as supportive evidence. Three separate extrapolations would be required, and none is justified. First, pulmonary arterial hypertension is classified as Group 1 pulmonary hypertension and is a primary pulmonary vascular disease characterized by precapillary obstructive remodeling of small pulmonary arteries with medial hypertrophy, intimal fibrosis, plexiform lesions, and in situ thrombosis occurring in the presence of normal pulmonary arterial wedge pressure. Left-sided heart failure produces Group 2 pulmonary hypertension, a postcapillary and hemodynamically distinct entity driven by passive backward transmission of elevated left atrial pressure, with or without a superimposed precapillary component. The cellular biology, natural history, and therapeutic responses of these two conditions diverge to the point that pulmonary vasodilator therapies effective in Group 1 disease are ineffective or harmful in Group 2 disease. Mechanistic observations derived from Group 1 pulmonary vascular remodeling therefore cannot be transferred to HFpEF or HFrEF. Second, that study measured total IgE, which behaves as a non-specific systemic marker of type 2 immune activation and is influenced by atopy, parasitic exposure, smoking, and a range of immune disorders, whereas the present review concerns Amb a 1-specific IgE, which reflects a defined antigenic memory; an association involving total IgE carries no implication for an allergen-specific response. Third, the cohort was small and the analysis cross-sectional, so reverse causation cannot be excluded, since advanced right-ventricular failure with hepatic congestion may itself alter immunoglobulin homeostasis. That association is accordingly cited here only as an illustration that the IgE axis has attracted attention in pulmonary vascular disease, and not as evidence bearing on Amb a 1-specific IgE in heart failure. Total IgE dynamics in primary pulmonary vascular disease must not be equated with Amb a 1-specific IgE in general heart failure populations. Amb a 1-specific IgE-positive HF patients with right-heart or pulmonary vascular vulnerability should therefore be prespecified as subgroups or interaction strata in future analyses rather than being pooled indiscriminately with all HF patients.

### 8.6. Fibrosis Hypothesis: Biologically Plausible but Not Proven for Amb a 1

The fibrosis hypothesis is mechanistically attractive but requires caution. IgE–FcεRI signaling has been linked to cardiac fibrosis in experimental models, and mast cell proteases can influence extracellular matrix turnover. However, Amb a 1-specific IgE is an antigen-specific marker; it is not equivalent to total IgE. A strong study would therefore test whether Amb a 1-specific IgE positivity is associated with fibrosis surrogates such as echocardiographic diastolic dysfunction, left atrial remodeling, cardiac MRI extracellular volume, galectin-3, soluble ST2, procollagen peptides, or longitudinal changes in NT-proBNP and troponin. Even then, causal inference would require temporal data and careful adjustment for age, hypertension, renal function, ischemic disease, diabetes, and obesity.

### 8.7. Therapeutic Implications: What Can Be Done Now and What Remains Investigational

At present, Amb a 1-specific IgE should not alter HF pharmacotherapy. Guideline-directed HF treatment remains based on established cardiology indications. The practical value, if validated, would be in comorbidity optimization and seasonal prevention: confirming genuine ragweed sensitization, treating rhinitis and asthma before pollen season, avoiding inappropriate sympathomimetic decongestants, improving inhaler technique, reducing exposure where feasible, and coordinating cardiology–allergology care. Allergen immunotherapy decisions should follow allergy guidelines and be individualized; HF status, beta-blocker use, frailty, asthma control, and anaphylaxis risk must be considered. Anti-IgE therapy is conceptually interesting but cannot be recommended for HF modification without dedicated trials.

It is useful to state concretely how a positive or negative Amb a 1-specific IgE result could alter clinical reasoning today, as distinct from what would require prospective validation. At present, the marker changes interpretation rather than prescription, and its practical value lies in four decisions that arise routinely in heart failure clinics.

The first is the attribution of seasonal dyspnea. When a patient with stable heart failure reports worsening breathlessness in late summer or early autumn, the immediate clinical question is whether this represents congestion requiring diuretic escalation or an extracardiac respiratory cause. Documented Amb a 1 sensitization in a patient whose symptoms cluster with local ragweed peaks raises the prior probability of an allergic contribution and supports a deliberate assessment of congestion using clinical examination, weight trend, natriuretic peptides, and, where available, lung ultrasound before treatment is intensified. Conversely, a negative Amb a 1 result in a patient with crude extract positivity argues against attributing symptoms to ragweed and redirects attention to cardiac or other respiratory causes. The clinical gain is diagnostic precision at the point of decision, and avoidance of diuretic escalation in patients who are not congested, in whom such escalation risks hypotension, renal deterioration, and electrolyte disturbance.

The second is comorbidity optimization with anticipatory timing. If genuine sensitization is confirmed, rhinitis and asthma treatment can be reviewed and intensified before the pollen season rather than reactively during it, using intranasal corticosteroids, appropriate inhaled therapy, and adherence review. This is a low-risk intervention in heart failure and addresses a modifiable contributor to symptom burden, sleep quality, and exercise tolerance.

The third is avoidance of harmful symptomatic self-treatment. Patients with allergic rhinitis frequently use over-the-counter sympathomimetic decongestants, which are undesirable in heart failure because of their pressor and arrhythmogenic effects, and may also use non-steroidal anti-inflammatory drugs for associated symptoms, which promote sodium retention and can precipitate decompensation. Identifying a sensitized patient creates the opportunity for explicit counseling on these agents and substitution with safer alternatives.

The fourth is the structuring of specialist referral and immunotherapy discussions. Molecular confirmation identifies which patients are plausible candidates for allergen immunotherapy, since ragweed immunotherapy targets Amb a 1-dominant responses, and equally identifies those in whom it would be inappropriate. Where immunotherapy is considered, heart failure severity, beta-blocker therapy, asthma control, frailty, and anaphylaxis risk must all be weighed, and beta-blockade in particular complicates the management of systemic reactions. These decisions belong to a joint cardiology and allergology assessment rather than to either specialty alone.

Two boundaries should be stated with equal clarity. Amb a 1-specific IgE must not be used to modify guideline-directed heart failure pharmacotherapy, infer cardiac causality, estimate prognosis, or justify anti-IgE therapy for cardiac indications, since none of these uses is supported by direct evidence. Its role at present is to sharpen symptom interpretation and comorbidity management in sensitized patients and define a phenotype worth studying prospectively. Whether the marker also carries prognostic or therapeutic implications is precisely the question that the study designs proposed in Section 8.3 and Section 10 are intended to answer.

### 8.8. Patient-Centered Management Implications

From the patient perspective, the main value of Amb a 1-specific IgE testing would not be to redefine heart failure diagnosis but to improve the interpretation of recurrent seasonal symptoms. Patients with HF frequently report dyspnea, fatigue, sleep disturbance, reduced activity, and anxiety related to symptom fluctuation. During ragweed season, these complaints may be amplified by allergic rhinitis, asthma, nasal obstruction, nocturnal cough, impaired sleep quality, or reduced outdoor activity. In sensitized patients, recognizing an allergic seasonal component could help clinicians distinguish congestion from respiratory symptom mimicry, avoid unnecessary diuretic escalation, optimize rhinitis and asthma treatment, and provide anticipatory counseling before peak pollen exposure. This approach is consistent with precision and patient-centered HF care because it integrates biological endotyping, environmental risk, comorbidity control, and patient-reported symptom burden.

## 9. Candidate Mechanisms and Measurements for Future Studies

Future studies should move beyond binary IgE positivity and integrate molecular allergy testing with HF phenotyping, pulmonary assessment, exposure science, and longitudinal outcomes. Table 3 summarizes candidate mechanisms and appropriate measurements.

## 10. Proposed Study Designs and Reporting Priorities

To move from plausibility to evidence, future studies should be designed around explicit clinical questions. A cross-sectional HF clinic study could compare Amb a 1-specific IgE-positive and -negative patients with respect to HF phenotype, allergic airway disease, NT-proBNP, echocardiographic parameters, eosinophils, and symptom burden. A prospective seasonal cohort could enroll HF patients before ragweed season and follow them through peak exposure, measuring symptoms, weight, NT-proBNP, lung function, medication changes, and healthcare use. A case-crossover design could evaluate whether high ragweed pollen days are followed by symptom escalation or HF presentations specifically among Amb a 1-specific IgE-positive patients. A mechanistic substudy could assess basophil activation, mast cell mediators, and fibrosis biomarkers in a deeply phenotyped subgroup.

Several design errors would substantially weaken this research area. Using crude ragweed extract IgE without Amb a 1-specific IgE would blur genuine sensitization and cross-reactivity. Measuring Amb a 1-specific IgE without total IgE would make it difficult to distinguish allergen-specific signal from global IgE elevation. Ignoring asthma, COPD, smoking, corticosteroid use, infection, pollution, and pollen season would create serious confounding. Using hospitalization alone as an endpoint without adjudicating congestion versus respiratory exacerbation would obscure the mechanism. Finally, claiming causality from cross-sectional associations would be methodologically inappropriate. A publishable clinical study should be precise, conservative, exposure-informed, and clinically anchored.

Table 4 summarizes the minimum and preferred reporting items that would make future clinical or translational manuscripts more reproducible, interpretable, and resistant to avoidable reviewer criticism.

## 11. Limitations

The main limitation of the current evidence base is the absence of direct clinical studies evaluating Amb a 1-specific IgE in established HF cohorts. Most cardiovascular evidence concerns total IgE, IgE receptors, mast cells, or broad allergic phenotypes rather than ragweed-specific molecular sensitization. Conversely, most ragweed evidence concerns allergic rhinitis, asthma, molecular diagnosis, or immunotherapy rather than HF. Environmental studies linking pollen to cardiovascular outcomes are heterogeneous and may be confounded by heat, air pollution, respiratory infections, medication adherence, healthcare-seeking behavior, and socioeconomic factors.

Consequently, the proposed model should be interpreted as a research framework. Its clinical value must be established through prospective, phenotype-rich studies rather than inferred from parallel literature. The distinction between sensitization, clinically active allergic disease, respiratory symptom mimicry, and objectively confirmed HF decompensation is essential. Until such data are available, Amb a 1-specific IgE should be considered a potential endotyping marker for research and comorbidity assessment, not a validated HF biomarker.

## 12. Conclusions

Amb a 1-specific IgE is a precise molecular marker of genuine ragweed sensitization, while the IgE-FcεRI–mast cell axis is increasingly relevant to cardiovascular inflammation and remodeling. Their intersection in HF is novel and biologically plausible but not yet clinically proven. The most defensible position is that Amb a 1-specific IgE may support HF endotyping, seasonal symptom interpretation, and cardio-allergology research in ragweed-endemic regions. It should not currently be used to modify HF pharmacotherapy, infer cardiac causality, or replace standard HF evaluation. Future studies should combine component-resolved diagnostics, pulmonary and environmental phenotyping, longitudinal HF outcomes, and conservative causal language to determine whether ragweed sensitization identifies a clinically meaningful and potentially modifiable subgroup of HF patients. Clinically, this framework may help physicians interpret seasonal dyspnea more accurately, integrate allergic comorbidity into HF assessment, and personalize preventive counseling for sensitized patients. Rather than replacing standard HF evaluation, Amb a 1-specific IgE testing may become useful as part of a broader patient-centered strategy combining cardiology, allergology, pulmonary assessment, and environmental exposure awareness.

## Figures and Tables

**Table 1:** Evidence domains relevant to Amb a 1-specific IgE in heart failure.

Evidence Domain	Representative Evidence	Relevance to Amb a 1-Specific IgE in HF	Interpretation
HF inflammation and fibrosis	Inflammation, microvascular dysfunction, cytokine activation, and extracellular matrix remodeling are central to HFpEF and HFrEF biology [5,6,7,8,9,10].	Provides a biologically plausible cardiac substrate in which immune signals could modify symptoms or remodeling.	Strong for HF biology; not proof of allergen-specific causality.
Total IgE and IgE–FcεRI axis	Experimental and clinical studies implicate IgE signaling in cardiac remodeling, fibrosis, atherogenesis, and mortality [11,12,13,14,15,16,17].	Supports cardiovascular relevance of the IgE pathway.	Moderate mechanistic evidence; total IgE findings must not be equated with Amb a 1-specific IgE.
Mast cell/cardiac interface	Cardiac mast cells release vasoactive, inflammatory, proteolytic, and profibrotic mediators [18,19,20,21].	Links allergic effector biology with myocardial, vascular, and right-heart-pulmonary mechanisms.	Important mechanistic bridge; needs validation in human HF cohorts.
Molecular allergology	CRD improves interpretation of genuine sensitization and cross-reactivity [22,23,24].	Justifies measuring Amb a 1-specific IgE rather than crude ragweed extract IgE alone.	Strong for allergy diagnostics; inferential for HF endotyping.
Ragweed and Amb a 1	Amb a 1 dominates ragweed IgE responses; European clinical and molecular studies support regional relevance [25,26,27,28,29,30,31,32,33].	Provides regional and molecular rationale for HF studies in ragweed-endemic areas.	Strong for ragweed allergy; no direct HF validation.
Pollen–cardiovascular interface	Studies link pollen and allergic airway disease with selected cardiovascular outcomes, but results are heterogeneous [36,37,38,39,40,41,42,43].	Supports seasonal-risk hypotheses and exposure-informed study designs.	Low-to-moderate and confounded; suitable for hypothesis generation only.

**Table 2:** Clinical interpretation matrix for Amb a 1-specific IgE in HF research and practice.

Finding	Most Defensible Interpretation	What Should Be Measured Next	What Should Not Be Concluded
Positive Amb a 1-specific IgE with rhinitis and/or asthma symptoms during the ragweed season	Genuine ragweed sensitization with clinically active seasonal disease.	Symptom scores, asthma control, spirometry or peak flow, NT-proBNP, congestion signs.	Do not conclude that Amb a 1 caused HF.
Amb a 1-specific IgE-positive without seasonal symptoms	Asymptomatic or subclinical sensitization; clinical impact is uncertain.	Exposure history, total IgE, comorbid atopy, repeat assessment during season.	Do not change HF therapy based on IgE alone.
High total IgE with Amb a 1-specific IgE-negative	Non-ragweed IgE biology or other causes of high total IgE.	Other aeroallergens, food IgE, smoking, parasites, atopic dermatitis, immune disease.	Do not label as ragweed-driven.
Ragweed extract IgE-positive with Amb a 1-specific IgE-negative	Possible cross-reactivity or sensitization to non-Amb a 1 components.	Amb a 8/9/10/11, profilin, CCD markers, clinical correlation.	Do not assume genuine Amb a 1 sensitization.
Seasonal HF symptom worsening during ragweed season	Potential trigger, mimic, or modifier requiring exposure-informed analysis.	Pollen, heat, PM_2.5_, infection, adherence, renal function, diuretic changes.	Do not attribute symptoms to allergy without component-resolved and clinical data.

**Table 3:** Candidate mechanisms and measurement strategy for Amb a 1-specific IgE studies in heart failure.

Mechanistic Hypothesis	Primary Measurements	Most Relevant HF Phenotype	Main Confounders	Best Endpoint
Atopic HF endotype	Amb a 1-specific IgE, ragweed extract IgE, total IgE, Amb a 8/9/10/11.	HFpEF and mixed-dyspnea cohorts.	Polysensitization, immunotherapy, smoking, parasitic disease.	Symptoms, quality of life, pulmonary–HF differentiation.
Seasonal ragweed exposure worsens symptoms	Daily pollen counts, symptom diary, weight, congestion score, NT-proBNP.	Recurrent seasonal dyspnea or decompensation.	Heat, PM_2.5_, infections, adherence, renal function.	Seasonal change in symptoms, NT-proBNP, congestion.
IgE effector activation contributes to inflammation	Eosinophils, basophil activation, tryptase, chymase, cytokines.	HF with allergic airway disease.	Corticosteroids, infections, mast cell disorders.	Inflammatory biomarker trajectory.
Allergic airway disease increases RV load	Spirometry, peak flow, oxygen saturation, RV function, pulmonary pressure.	RV dysfunction, PH, COPD/asthma overlap.	COPD, sleep apnea, pulmonary embolism.	RV function, exercise tolerance.
IgE axis relates to fibrosis/remodeling	Echocardiography, cardiac MRI, ECV, galectin-3, ST2, PICP/PIIINP.	HFpEF, hypertensive HF, nonischemic cardiomyopathy.	Age, CKD, hypertension, ischemic disease.	Longitudinal fibrosis surrogate progression.

**Table 4:** Reporting priorities for clinical or translational studies of Amb a 1-specific IgE in heart failure.

Item	Minimum Requirement	Preferred Standard	Rationale
HF phenotype	LVEF, NYHA class, NT-proBNP, HF etiology.	Guideline-compatible HF category, echocardiography, renal function, comorbidity index.	Prevents phenotype mixing.
IgE assessment	Amb a 1-specific IgE platform, units, threshold.	Total IgE, ragweed extract IgE, Amb a 8/9/10/11, assay limits, season of sampling.	Prevents sensitization misclassification.
Allergic disease	Rhinitis/asthma history.	Validated symptom scores, asthma control, spirometry/peak flow, medication use.	Separates sensitization from active disease.
Exposure	Season at sampling.	Daily pollen, weather, PM_2.5_/NO_2_/ozone, local exposure metrics.	Tests trigger and seasonality hypotheses.
Endpoints	Symptoms or hospitalization.	Adjudicated HF decompensation versus respiratory exacerbation, NT-proBNP trajectory, quality of life.	Improves clinical interpretability.
Statistics	Adjustment for major confounders.	Prespecified interactions by HF phenotype, asthma/COPD, RV dysfunction, season.	Reduces residual confounding and overstatement.
Causal language	Avoid causal claims in cross-sectional work.	Use mechanistic, longitudinal, or associative wording matched to the design.	Protects scientific validity.

## Data Availability

No new data were created or analyzed in this study. Data sharing is not applicable to this article.

## Abbreviations

The following abbreviations are used in this manuscript:

Amb a 1	Ambrosia artemisiifolia major allergen 1
CCD	Cross-reactive carbohydrate determinant
CD23	Low-affinity immunoglobulin E receptor
CKD	Chronic kidney disease
COPD	Chronic obstructive pulmonary disease
CRD	Component-resolved diagnosis
ECM	Extracellular matrix
ECV	Extracellular volume
FcεRI	High-affinity immunoglobulin E receptor
HF	Heart failure
HFimpEF	Heart failure with improved ejection fraction
HFmrEF	Heart failure with mildly reduced ejection fraction
HFpEF	Heart failure with preserved ejection fraction
HFrEF	Heart failure with reduced ejection fraction
IgE	Immunoglobulin E
IL	Interleukin
LVEF	Left ventricular ejection fraction
miR	microRNA
MRI	Magnetic resonance imaging
NO_2_	Nitrogen dioxide
NT-proBNP	N-terminal pro-B-type natriuretic peptide
NYHA	New York Heart Association
PH	Pulmonary hypertension
PICP	Procollagen type I C-terminal propeptide
PIIINP	Procollagen type III N-terminal propeptide
PM_2.5_	Particulate matter with an aerodynamic diameter ≤2.5 μm
QoL	Quality of life
RAAS	Renin–angiotensin–aldosterone system
RV	Right ventricular
ST2	Suppression of tumorigenicity 2
TGF-β	Transforming growth factor beta
